# *Actinobacillus* utilizes a binding protein–dependent ABC transporter to acquire the active form of vitamin B_6_

**DOI:** 10.1016/j.jbc.2021.101046

**Published:** 2021-08-04

**Authors:** Chuxi Pan, Alexandra Zimmer, Megha Shah, Minh Sang Huynh, Christine Chieh-Lin Lai, Brandon Sit, Yogesh Hooda, David M. Curran, Trevor F. Moraes

**Affiliations:** Department of Biochemistry, University of Toronto, Toronto, Ontario, Canada

**Keywords:** pyrodoxial-5-phosphate uptake, protein transporters, periplasmic binding protein, Gram-negative bacteria, AfuABC, *Actinobacillus* ferric uptake ABC, BPDT, binding protein–dependent transporter, DXP, deoxyxylulose 5-phosphate, G6P, glucose-6-phosphate, GdHCl, guanidine hydrochloride, ITC, isothermal titration calorimetry, MR, molecular replacement, MST, microscale thermophoresis, P5P, pyridoxal-5′-phosphate, P5PA, P5P binding protein A, PBST, PBS with 0.05% Tween-20, PDB, Protein Data Bank, PL, pyridoxal, PLP, pyridoxal phosphate, PM, pyridoxamine

## Abstract

Bacteria require high-efficiency uptake systems to survive and proliferate in nutrient-limiting environments, such as those found in host organisms. ABC transporters in the bacterial plasma membrane provide a mechanism for transport of many substrates. In this study, we examine an operon containing a periplasmic binding protein in *Actinobacillus* for its potential role in nutrient acquisition. The electron density map of 1.76 Å resolution obtained from the crystal structure of the periplasmic binding protein was best fit with a molecular model containing a pyridoxal-5′-phosphate (P5P/pyridoxal phosphate/the active form of vitamin B_6_) ligand within the protein's binding site. The identity of the P5P bound to this periplasmic binding protein was verified by isothermal titration calorimetry, microscale thermophoresis, and mass spectrometry, leading us to name the protein P5PA and the operon P5PAB. To illustrate the functional utility of this uptake system, we introduced the P5PAB operon from *Actinobacillus pleuropneumoniae* into an *Escherichia coli* K-12 strain that was devoid of a key enzyme required for P5P synthesis. The growth of this strain at low levels of P5P supports the functional role of this operon in P5P uptake. This is the first report of a dedicated P5P bacterial uptake system, but through bioinformatics, we discovered homologs mainly within pathogenic representatives of the Pasteurellaceae family, suggesting that this operon exists more widely outside the *Actinobacillus* genus.

Binding protein–dependent transporter (BPDT) systems are composed of three components: a periplasmic binding protein and a permease–ATPase complex embedded in the inner membrane. The periplasmic binding protein sequesters a specific substrate and recruits it to the permease–ATPase plasma membrane complex, which uses ATP hydrolysis to induce substrate influx from the periplasm into the cytosol (1).

A wide variety of substrates have been identified for BPDT systems, including zinc, iron, amino acids, sugar, as well as vitamins (2, 3). BtuCDF is one of the well-characterized vitamin BPDT systems that is specifically dedicated for importing vitamin B_12_. Vitamin B_12_ belongs to the B-type vitamin group that is highly water soluble and contributes significantly to enzymatic catalysis as cofactors. However, among the B vitamins, transporters specific for vitamin B_6_ remained under investigation (4).

Vitamin B_6_ has multiple chemical forms, including pyridoxal (PL), pyridoxamine (PM), pyridoxine, and their corresponding phosphorylated products. However, the biologically active form of vitamin B_6_ is pyridoxal-5′-phosphate (P5P/pyridoxal phosphate [PLP]) (5, 6). Vitamin B_6_ functions as an essential cofactor for a variety of cellular catalytic reactions and nutrient metabolisms, including amino acids, fatty acids, carbohydrates (5), and is also implicated in regulating cell stress and oxidants (6, 7). In most microorganisms, approximately 1.5% of genes encode vitamin B_6_-dependent enzymes, which are responsible for catalyzing more than 140 biochemical reactions (5, 8). Vitamin B_6_ deficiency in *Helicobacter pylori* interferes with the motility appendage synthesis and the establishment of chronic colonization in mice leading to the attenuated virulence (9). Moreover, vitamin B_6_ also plays a significant role in the growth and survival of *Mycobacterium tuberculosis* (10). Thus, acquiring vitamin B_6_ is critical for prokaryotic survival.

*Actinobacillus pleuropneumoniae* is an important Gram-negative bacterial pathogen that preferentially infects and colonizes the lower respiratory tracts of pigs. It is the causative pathogen for the highly contagious porcine pleuropneumonia, which is characterized by pulmonary edema, hemorrhage, and necrosis (11, 12). Acute outbreaks of *A. pleuropneumoniae* infection can result in swine death within 24 h, leading to high economic losses for the swine industry (11, 13). Previous studies have identified nutrient acquisition pathways as virulence factors used by *A. pleuropneumoniae* to acquire essential nutrients (12). Thus, efficient nutrient acquisition systems, such as binding BPDTs, have evolved in *A. pleuropneumoniae* and provide it with the necessary nutrients to outgrow competing organisms.

Despite playing an important role in nutrient acquisition, BPDT systems in *A. pleuropneumoniae* have either been misannotated or gone completely uncharacterized. For example, the BPDT system designated *Actinobacillus* ferric uptake ABC (AfuABC) was identified in *A. pleuropneumoniae* and, based on sequence homology to FbpA, the periplasmic binding protein AfuA was proposed to be a transporter for ferric iron (14). However, the crystal structure of AfuA in complex with glucose-6-phosphate (G6P) revealed that AfuABC system is a highly specific transporter for hexose phosphate/heptose phosphate molecules and provides a significant alternative carbon source for bacterial metabolism in the lower gut (15). Following the identification of the role of AfuA, we discovered another gene (APJL_RS02700) in *A. pleuropneumoniae* with high sequence identity to AfuA and began to characterize the unknown structure and function of this putative periplasmic binding protein.

In this study, we determine the structure of the periplasmic binding protein encoded by APJL_RS02700 in complex with a ligand and identify that ligand to be PLP/P5P (vitamin B_6_). We confirm the identity of the ligand in the binding pocket using mass spectrometry and further show, using isothermal titration calorimetry (ITC) and microscale thermophoresis (MST), that P5P binds this periplasmic binding protein with high affinity. Finally, we demonstrate that expressing the operon containing APJL_RS02700 in a strain of *Escherichia coli* deficient in vitamin B_6_ synthesis rescues its growth in media containing low levels of P5P. We propose the name P5PA, for P5P binding protein A for this newly characterized periplasmic binding protein. To our knowledge, P5PA is the only bacterial protein shown to promote uptake of the active form of vitamin B_6_.

## Results

### The structure of APJL_RS02700 reveals an endogenously bound ligand, P5P

Whilst characterizing the function of AfuA (15), we discovered another gene in *A. pleuropneumoniae* (APJL_RS02700) with high sequence identity (54%) to AfuA. To determine the function of this homologous protein, we cloned, expressed, and purified it recombinantly in *E. coli*.

**Figure 1:**
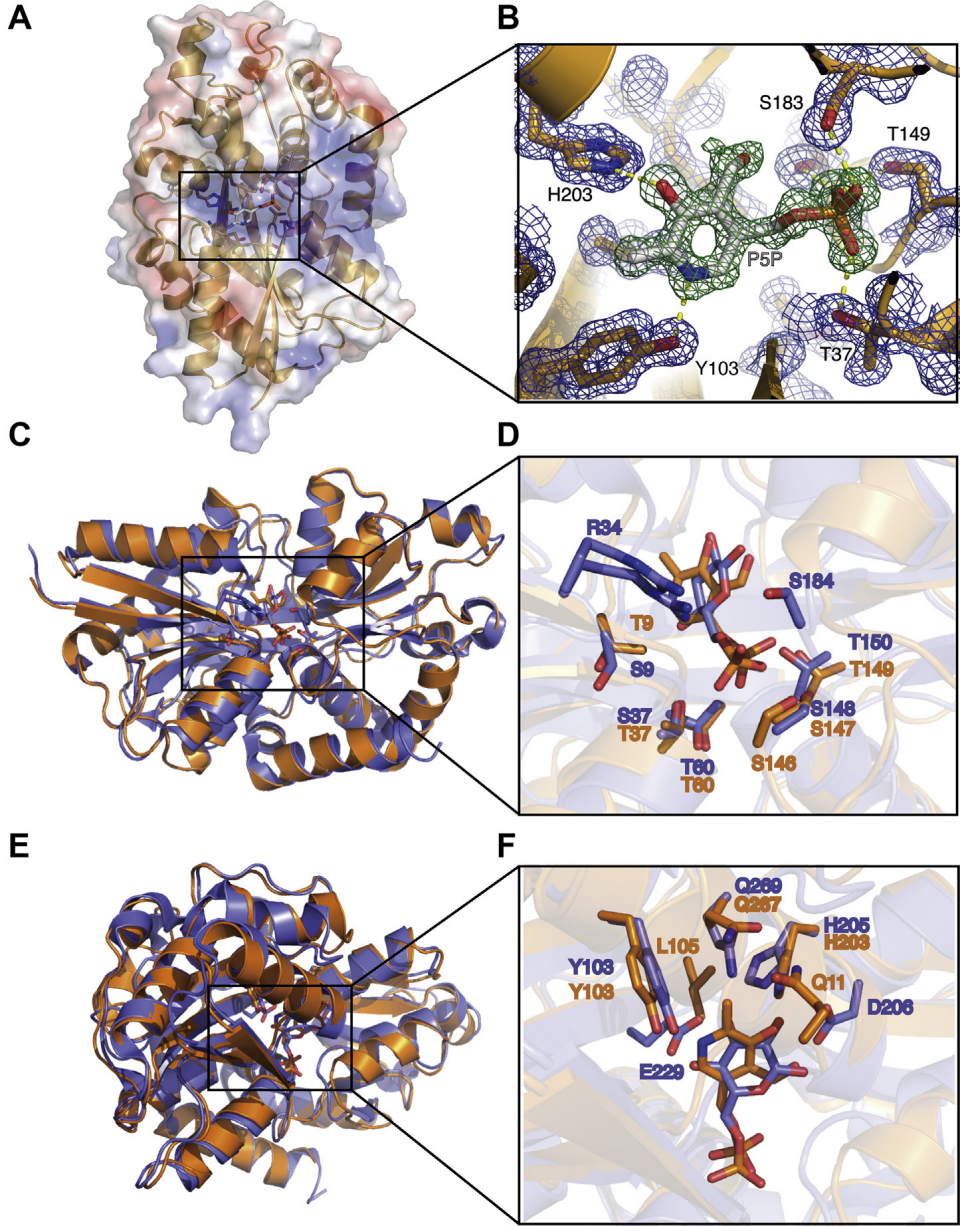
**P5PA is a type II periplasmic binding protein with a P5P binding site.***A*, ribbon diagram of P5PA (*green*) bound to endogenously copurified vitamin B_6_ (P5P, *magenta*) (Protein Data Bank code: 6WCE determined in this study). *B*, the copurified P5P is shown in the *inset* with interacting residues shown as *sticks*. The pyridine ring of P5P (vitamin B_6_) is coordinated by Y103 and H203, whereas the phosphate group are stabilized together by T37, G148, T149, and S183. 2*F*o–*F*c electron density map (*blue mesh*) is shown for the P5PA protein that surrounds the copurifying ligand that is modeled into an *F*o–*F*c omit map (*green mesh* contoured at three sigma). Overlaid structures of P5PA (*orange*) and AfuA (*blue*) (Protein Data Bank code: 4R73). *C* and *D*, the key residues coordinating the phosphate moieties of G6P and P5P in AfuA and P5PA, respectively. *E* and *F*, the residues stabilizing the ring moieties of G6P and P5P in AfuA and P5PA, respectively. P5PA, P5P binding protein A.

The purified protein was crystalized and subjected to X-ray crystallographic characterization. Using molecular replacement (MR) with AfuA as a model, the structure of “APJL_RS02700” was solved and refined to a resolution of 1.75 Å (see Experimental procedures section and Table 1). The resulting high-resolution structure revealed a typical type II periplasmic binding protein with two β-strands connecting two globular domains. However, the binding cleft located above the β-strands at the interface of the two globular domains (Fig. 1*A*) revealed clear positive density indicating a possible copurifying ligand (Fig. 1*B*). An *in silico* screen for possible ligands revealed that neither G6P nor fructose-6-phosphate, the two primary ligands that bind AfuA, fit the positive density in the binding pocket. Further *in silico* screening of potential ligands containing six-member rings and phosphate groups indicated that the active form of vitamin B_6_, called PLP/P5P, best fit in the unmodeled electron density (Fig. 1*B*). Thus, we named APJL_RS02700, P5PA for P5P binding protein A.

**Table 1:** Data collection and refinement statistics for P5PA

PDB: 6WCE	A.pl P5PA—vitamin B_6_
Data collection	
Resolution (Å)	35.69–1.75 (1.82–1.75)^a^
Space group	P 1 2_1_ 1
Unit cell	
a (Å)	51.47
b (Å)	42.27
c (Å)	69.60
α (°)	90
β (°)	106.82
γ (°)	90
Total reflections	50,884 (1986)
Unique reflections	26,906 (1675)
Multiplicity	1.9 (1.2)
Completeness (%)	92.8 (59.0)
I/σI	14.34 (3.68)
Wilson *B*-factor	7.94
*R*_merge_	0.045 (0.29)
CC_1/2_	0.991 (0.794)
CC∗	0.998 (0.941)
Refinement	
*R*_work_ (%)	16.1
*R*_free_ (%)	19.5
Number of non-H atoms	2711
Protein atoms	2454
Ligand	16
Solvent	241
RMSD	
Bond lengths (Å)	0.007
Bond angles (°)	1.16
Average *B*-factors (Å^2^)	
Overall	9.54
Proteins	8.94
Ligand	6.49
Solvent	15.87
Ramachandran plot^b^	
Favored region (%)	99
Allowed region (%)	1
Outliers (%)	0.00

a Highest-resolution shell is provided in parenthesis.

b Ramachandran plot statistics were obtained from the Worldwide PDB X-ray structure validation report.

**Figure 2:**
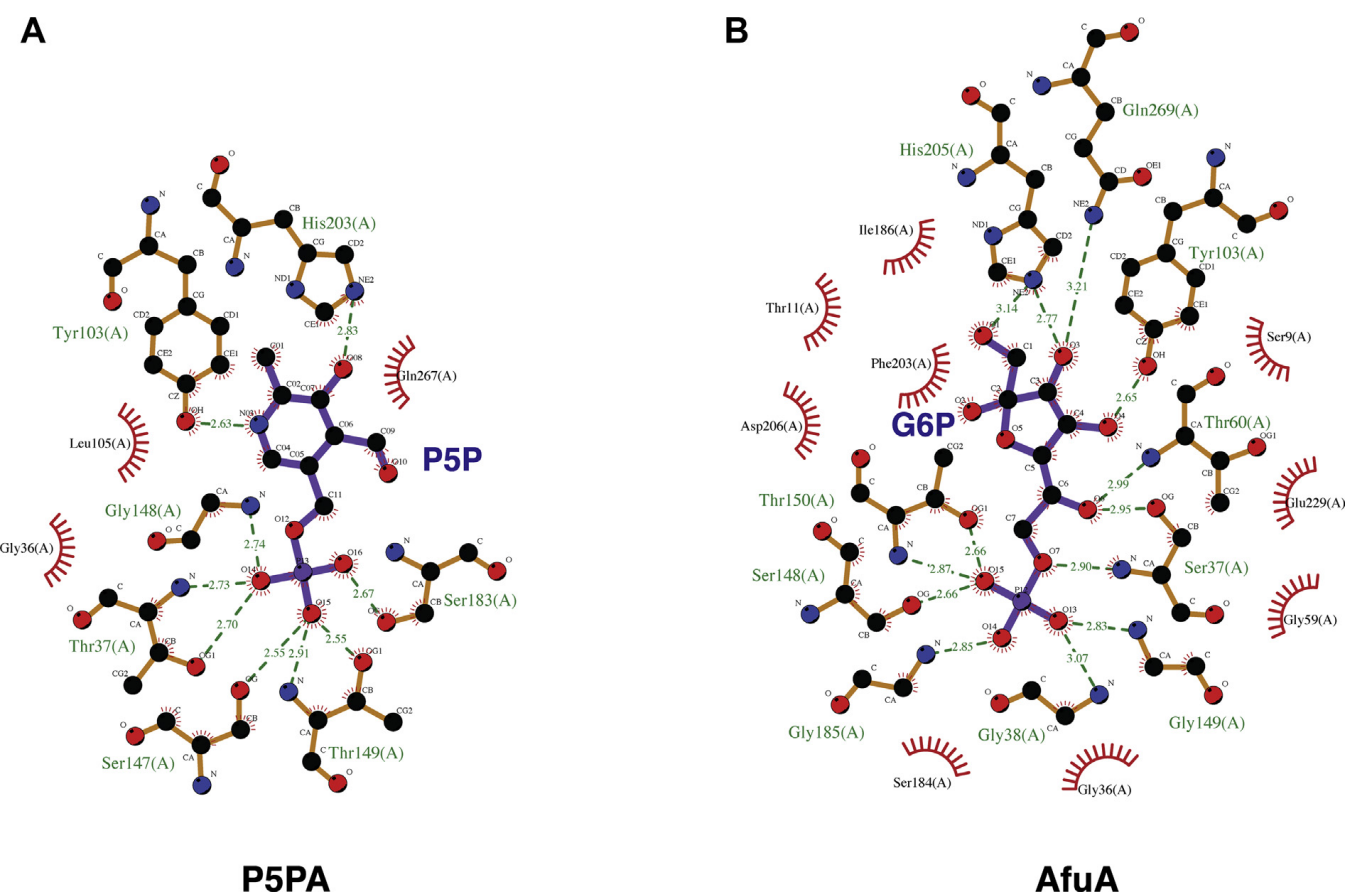
**Residues involved in the ligand coordination within the binding cleft.***A*, binding cleft of P5P. *B*, binding cleft of AfuA. *Green dash lines* represent hydrogen bonds with the bond length values. *Red eyelashes* represent hydrophobic interactions. The figure was generated by LigPlot (51).

The model of P5PA with P5P in the binding pocket reveals a network of hydrogen bonds and hydrophobic clefts that primarily hold the pyridine ring and phosphate group of P5P in place. While Tyr103 interacts with the pyridine ring nitrogen and His203 coordinates the pyridine hydroxyl group, the phosphate group of P5P is stabilized by collective interactions with the side chains of Thr37, Ser147, Thr149, and Ser183 and the backbones of Thr37, Thr149, and Gly148 (Figs. 1*A* and 2*A*). In addition, the dipole created the N terminus of three alpha helices beginning at residues Thr37, Gly148, and Ser183, respectively, and create a positive environment for the negatively charged phosphate on P5P (Fig. 1, *B* and *C*). Moreover, the hydrophobic interactions with Gly36, Leu105, and Gln267 contributed to retaining the P5P molecule within this binding pocket (Fig. 2*A*).

P5PA and AfuA share 54% sequence identity as well as structural similarity (RMSD = 0.793). However, they are involved in transporting different small nutrient molecules. AfuA plays an important role in transporting G6P, which has a sugar ring and a phosphate moiety, whereas P5PA is responsible for taking up vitamin B_6_, which has a pyridine ring as well as a phosphate moiety. Comparing the two binding clefts, it becomes clear that the phosphate moieties of G6P and P5P are both coordinated *via* hydrogen bonds formed with side chains and backbones of glycine, serine, and threonine (Figs. 1, *C* and *D* and 2, *A* and *B*). In the binding pocket of AfuA, Ser37, Gly38, Thr60, Ser148, Gly149, Thr150, and Gly185 surround the phosphate group of G6P. Similarly, the phosphate moiety of P5P is enclosed by Thr37, Ser147, Gly148, Thr149, and Ser183 in the binding site of P5PA. The ring moieties of G6P and vitamin B_6_ are also stabilized by the similar intermolecular forces (Figs. 1, *E* and *F* and 2, *A* and *B*). In the binding site of AfuA, the sugar ring is coordinated by forming hydrogen bonds with Tyr103, His205, and Gln269. Likewise, in the binding cleft of P5PA, the pyridine ring of P5P interacts with Tyr103 and His203. However, more hydrophobic interactions are present in the binding cleft of AfuA to seize G6P, including Ser9, Thr11, Gly36, Gly59, Ser184, Ile186, Phe203, Asp 206, and Glu229, whereas only Gly36, Leu105, and Gln267 participate in the hydrophobic network surrounding P5P in the binding pocket of P5PA. It was previously reported that H205A, D206A, or E229A mutants of AfuA have undetectable binding affinity for G6P, implying the loss of capacity to sequester G6P (15). However, no hydrophobic interactions were shown between His203 and Gln267 in the binding pocket of P5PA (Fig. 2*A* and Fig. S1), which thus cannot offer the binding determinants to accommodate G6P. It is possible that the substrate specificity of P5PA could be modified to accommodate G6P by making A205D and A228E mutations in addition to modifying Gln267—the major contacting residue differences between P5PA and AfuA. Our structural analysis of P5PA suggests that it is a highly specific transporter for P5P and does not possess the capacity to transport G6P because of a lack of hydrophobic interactions required to mediate binding of G6P.

### Identification of P5PA by mass spectrometry

**Figure 3:**
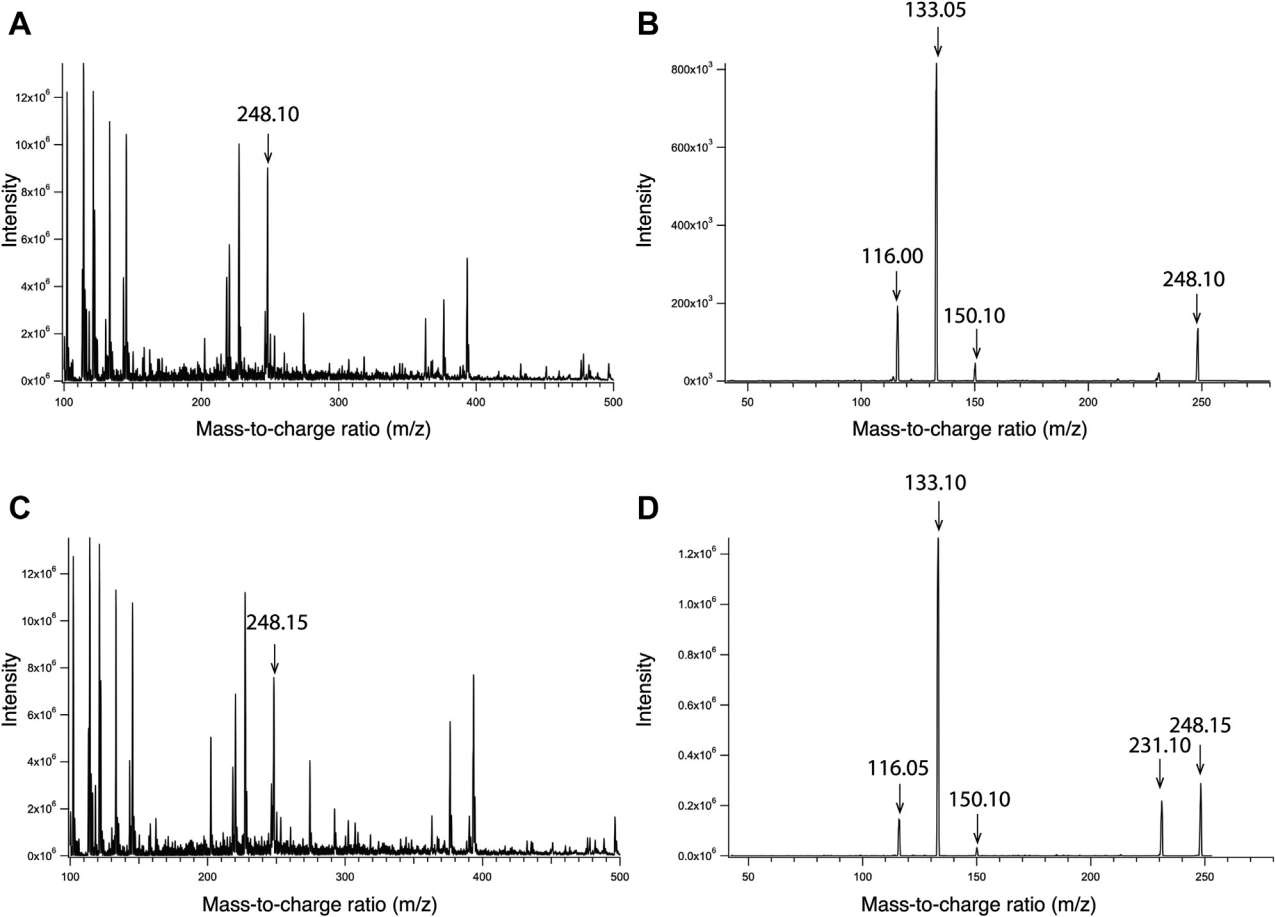
**LC–MS/MS profiles of vitamin B**_**6**_**solution and holo-P5PA extract.** Holo-P5PA was purified and treated with acidic acetone to denature and precipitate the protein. The soluble extract, which contained the endogenous ligand, was harvested. Acetonitrile and saturated NH_4_Cl were applied to remove Tris and NaCl, and the extract was then subjected to LC–MS/MS. The dissolved vitamin B_6_ (P5P) solution was used as a positive control. The presence of P5P was indicated by a peak at 248 *m*/*z*. *A*, LC–MS/MS profile of the P5P solution from 100 to 500 *m*/*z*. *B*, the fragmented P5P solution peak at 248 *m*/*z*. *C*, LC–MS/MS profile of the extract from 100 to 500 *m*/*z*. *D*, the fragmented extract peak at 248 *m*/*z*. P5PA, P5P binding protein A.

To confirm that vitamin B_6_ is a ligand of P5PA and determine if P5PA has any other potential copurifying substrates, we extracted any copurifying ligands that were endogenously bound to P5PA and performed LC–MS/MS (Fig. 3). The molecular weight of vitamin B_6_ (P5P) is 247 Da. As a proton was added to ionize P5P, the *m*/*z* representing P5P became 248. We used P5P powder dissolved in ultrapure water as a positive control. The peak representing 248 *m*/*z* was detected in both the positive control P5P solution and the ligands extracted from P5PA, although the peak in the P5P solution had higher intensity (Table 2 and Fig. 3, *A* and *C*). In order to identify the molecule represented by the detected 248 *m*/*z* peak, the products at 248 *m*/*z* were fragmented by nitrogen gas. The expected transition of 248 → 150 *m*/*z* can be used to characterize P5P molecules (16). The 248 → 150 *m*/*z* fragmentation pattern was observed in both the extract (Fig. 3*D*) and P5P solution (Fig. 3*B*), implying that P5P was present in the extract of ligand-bound P5PA. Overall, the major peaks present in the P5P solution and the extract had the same *m/z* but different intensities, suggesting that no other P5P derivatives were identified by LC–MS/MS.

**Table 2:** LC–MS analysis of ligands extracted from holo-P5PA and dissolved P5P solution

Sample	Original concentration (μM)	248 *m*/*z* intensity
P5P solution	236	9 × 10^6^
Substrates extracted from holo-P5PA	236	7.8 × 10^6^

### Measuring the binding affinity between P5PA and vitamin B_6_ vitamers using ITC and MST

**Figure 4:**
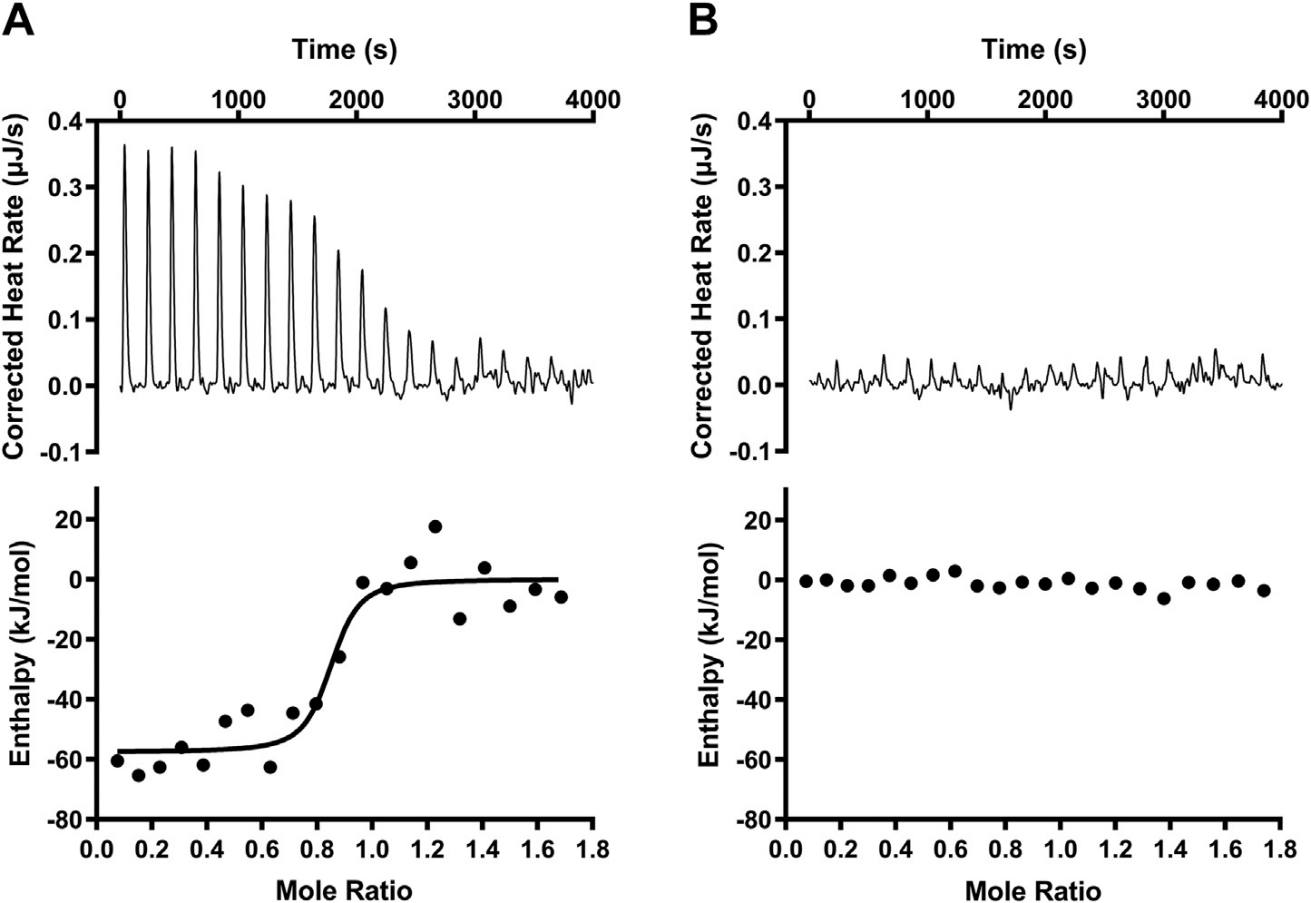
**P5PA has a strong and specific interaction with P5P.***A*, representative ITC data showing the interaction between P5PA and P5P. About 45 μM of P5PA was titrated with 300 μM of P5P. The thermodynamic parameters were measured by an ITC-200. *B*, ITC data showing the titration of G6P into P5PA. The parameters were the same as (*A*). ITC, isothermal titration calorimetry; P5PA, P5P binding protein A.

To verify whether P5PA binds P5P and serves as a component of a high-affinity BPDT system, we first examined the affinity between the two molecules using ITC. We determined a dissociation constant (*K*_*d*_) of 36 ± 7 nM, which suggests that there is a strong interaction between P5PA and P5P (Table 3 and Fig. 4*A*). This tight binding activity was further supported by MST results with *K*_*d*_ = 28 nM (Table 3 and Fig. S2).

**Table 3:** Dissociation binding constants (*K*_*d*_) for the interaction between P5PA, mutants, and P5P (by ITC and MST)

Protein	*K*_*d*_ (μM)	Method
P5PA WT	0.036 ± 0.007	ITC
P5PA WT	0.028 ± 0.015	MST
P5PA Y103A	17 ± 8	
P5PA T149A	1.3 ± 0.3	
P5PA H203A	10 ± 4	

Biological replicates of n = 3 were completed.

To illustrate that residues involved in P5P coordination are critical in binding, three site-directed mutations were introduced into the binding cleft of P5PA. While all three mutants (Y103A, T149A, and H203A) exhibited an impaired binding capacity to P5P (Table 3 and Fig. S2), the pyridine ring–related mutants Y103A and H203A showed 10× lower affinities than that of T149A.

As P5P belongs to vitamin B_6_ that contains multiple vitamers with similar chemical structures, we queried whether P5PA had other potential ligands and was able to bind other vitamin B_6_ vitamers. We added P5PA to another two vitamin B_6_ vitamers: PL and PM. The MST-determined *K*_*d*_ for PL and PM was 13 and 8 mM, respectively (Table 4 and Fig. S3), indicating weak interactions with these two molecules and demonstrating that P5PA is a highly specific binding protein for P5P.

**Table 4:** Dissociation binding constants (*K*_*d*_) for the interaction between P5PA and different vitamin B_6_ vitamers

Compound	*K*_*d*_
P5P	28 ± 15 nM
PL	13 ± 4 mM
PM	8 ± 4 mM
G6P	11 ± 3 mM

Fluorescently labeled P5PA was mixed with a serial dilution of P5P, PL, PM, and G6P, respectively. Fluorescence changes and *K*_*d*_ were measured and analyzed by MST. Biological replicates of n = 3 were completed.

As P5PA is a homolog of AfuA, we wondered whether P5PA is able to interact with hexose phosphate molecules. We measured the interaction between G6P and P5PA using ITC. The released heat was approximately 3 kJ/mol for each G6P to P5PA titration, which was insufficient to calculate a dissociation constant (Fig. 4*B*). This reflects no significant binding activity between G6P and the periplasmic binding protein P5PA supporting our structural analysis that AfuA and P5PA are components of distinct specific nutrient uptake systems.

### P5PA facilitates *E. coli* growth at low levels of vitamin B_6_

Since our biochemical results reveal that P5P is a ligand of P5PA *in vitro*, we wondered whether the gene cluster containing P5PA would be able to functionally uptake P5P and thus complement a vitamin B_6_ deficiency in *E. coli*. In WT *E. coli* (strain K-12), the active form of vitamin B_6_ (P5P) can be synthesized from d-erythrose 4-phospate, pyruvate, and glyceraldehyde 3-phosphate by enzymes encoded by *pdx* family genes (Fig. S4). When the vitamin B_6_ biosynthesis pathway is disrupted, *E. coli* K-12 displays severe growth defects in the absence of exogenous P5P (17). The *E. coli* K-12 auxotroph, Δ*pdxB*, contains a deletion of 4-phosphoerythronate dehydrogenase that plays a significant role in converting d-erythrose 4-phosphate to P5P.

We tested K-12::Δ*pdxB* for its growth capacity in M9 minimal media supplemented with P5P at different concentrations varying from 0 to 10 μM. After 24 h of incubation, we found that the WT strain of *E. coli* K-12 grew to stationary phase and that its growth was not affected by the concentration of supplemented P5P. However, the Δ*pdxB* mutant had severe growth deficiency in all conditions except P5P concentrations of 5 and 10 μM (Fig. S5). As the Δ*pdxB* mutant showed a measurable growth deficiency at low concentrations of P5P, it was used to investigate the role of P5PA.

P5PA is encoded by the *p5pAB* operon in *A. pleuropneumoniae*. BPDT operons typically encode a periplasmic binding protein, a permease with transmembrane domains, and an ATPase. However, there was no gene encoding an ATPase component downstream of *p5pAB*. Therefore, we have two hypotheses to explain how P5PAB functions. The first hypothesis is that the gene annotated as “*p5pB*” encodes both the transmembrane domains and ATPases. The permeases in BPDT systems have been associated with Pfam domains PF00664 and PF00528, whereas the ATPase activity has been associated with domain PF00005. They are relatively common in *A. pleuropneumoniae*, and a search of the proteome yielded 31 proteins with transmembrane domains and at least 74 ATPases, though many of the latter are likely to be cytosolic. The *p5pB* gene was found to contain the PF00528 transmembrane domain but not the ATPase domain. The downstream region contains an apparently pseudogenized TonB-dependent transporter 293 bp downstream of *p5pB* and another pseudogene 322 bp further downstream. Interestingly, that second pseudogene contains both transmembrane and ATPase domains. It was also detected in a nontruncated form in approximately half of the available *A. pleuropneumoniae* genomes and further was detected in a nontruncated form in eight other species of *Actinobacillus* and 24 species from related genera. The significant prevalence of the pseudogenized form within *A. pleuropneumoniae* suggests that it is no longer useful and is in the process of being lost, though it is enticing to consider that at one point in time it may have had a relevant function because of the prevalence in other species and proximity to the *p5pAB* operon.

The other hypothesis to explain the functionality is that the *p5pAB* operon shares ATPases with other BPDT systems. Other studies have demonstrated that the ATPase component MsiK is shared by a couple of ABC transport systems for disaccharides in Gram-positive bacteria (18, 19). As P5PA shares 54% amino acid sequence identity with its homolog AfuA and P5PB shares 60% identity with AfuB, we speculated that P5PAB may be able to utilize the ATPase component, AfuC, from the AfuABC hexose-phosphate uptake system. Thus, we made two constructs, one containing the *p5pAB* operon and another containing the *p5pAB* operon together with *afuC*.

**Figure 5:**
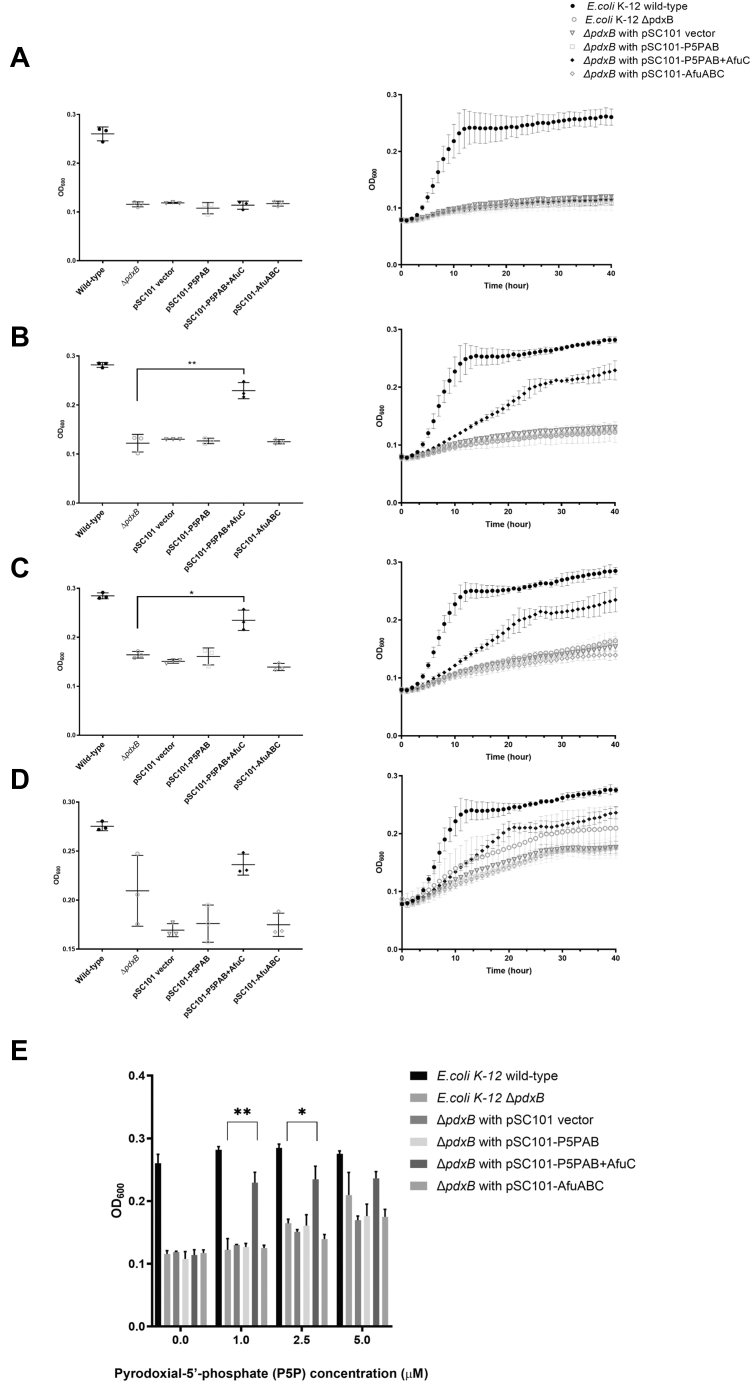
***p5pAB + afuC* restored the growth rate of *Escherichia coli* K-12 Δ*pdxB* mutant.***E. coli* K-12 Δ*pdxB* mutants were transformed with four constructs: pSC101, pSC101-P5PAB, pSC101-P5PAB + AfuC, and pSC101-AfuABC. They were then incubated in 200 μl of M9 supplemented with P5P at (*A*) 0 μM, (*B*) 1 μM, (*C*) 2.5 μM, and (*D*) 5 μM. The starting absorbance at 600 nm was 0.08, and reading of an absorbance at 600 nm was measured every 15 min during a time course of 40 h. The hourly absorbance at 600 nm data points are reported in the graph. *E*, a bar graph representing the mean absorbance at 600 nm readings after 40 h. The error bars represent standard deviations from n = 3 transformants. (∗*p* < 0.05 and ∗∗*p* < 0.01).

In order to rescue growth of the Δ*pdxB* mutant, this strain was transformed with either pSC101 empty vector, pSC101-P5PAB, pSC101-P5PAB + AfuC, or pSC101-AfuABC and grown in M9 media supplemented with vitamin B_6_ (Fig. 5). When P5P was supplied at concentrations of 1 and 2.5 μM, growth of the Δ*pdxB* mutant was rescued by the expression of pSC101-P5PAB + AfuC (*p* < 0.01, two-tailed *t* test); however, expression of pSC101, pSC101-P5PAB, and pSC101-AfuABC did not have any effect on the growth of the Δ*pdxB* mutant (Fig. 5*E*). When P5P was supplied at 5 μM, the growth defects of the four transformants and the Δ*pdxB* mutant were partially restored, whereas the pSC101-P5PAB + AfuC transformant grew the fastest of all the transformants to stationary phase (Fig. 5*D*). Furthermore, the expression of the pyridine ring–related P5PA–binding mutants (Y103A and H203A) did show a growth defect compared with WT P5PA when placed within the pSC101-P5PAB + AfuC growth assay (Fig. S6). Given our results, we conclude that P5PAB can acquire exogenous P5P but requires a shared ATPase component from other ABC transport systems. In this case, the ATPase AfuC supplies sufficient energy to allow for P5P uptake. This gain-of-function experiment in *E. coli* verifies that P5PA together with P5PB performs as a high-affinity vitamin B_6_ (P5P) uptake system.

### P5PA are conserved in pathogenic strains of *Pasteurellaceae* family

In order to determine whether this vitamin B_6_ transporter is conserved in microorganisms other than *A. pleuropneumoniae*, we searched for homologs using the P5PA amino acid sequence as a template. A sequence was denoted homologous when it was found to share a minimum of 65% amino acid identity with P5PA. To further validate that our predicted P5PA hits were not the G6P-binding AfuA, we collected the sequences identified by BLAST outside the Pasteurellaceae sharing >40% sequence identity over >80% of any P5PA seed sequence. There were 471 sequences meeting this threshold but none that met our P5PA threshold of >65% identity. These 471 sequences were then searched for the P5PA sequence motifs identified by MEME. None fit the motif pattern consistent with P5PA, 468 were consistent with the AfuA motif pattern (Fig. S7), and three were missing any motifs at all. Within the unique meme logo analysis for P5PA *versus* AfuA, there is only one residue Gln267 within P5PA that makes direct contact with P5P, and it is the positioning of the surrounding helices and hydrophobic residues including Leu105 and Gly36 that create the ideal environment for binding a phosphorylated pyridine ring containing P5P molecule. This meme analysis importantly suggests that P5PA is only found within the Pasteurellaceae.

**Figure 6:**
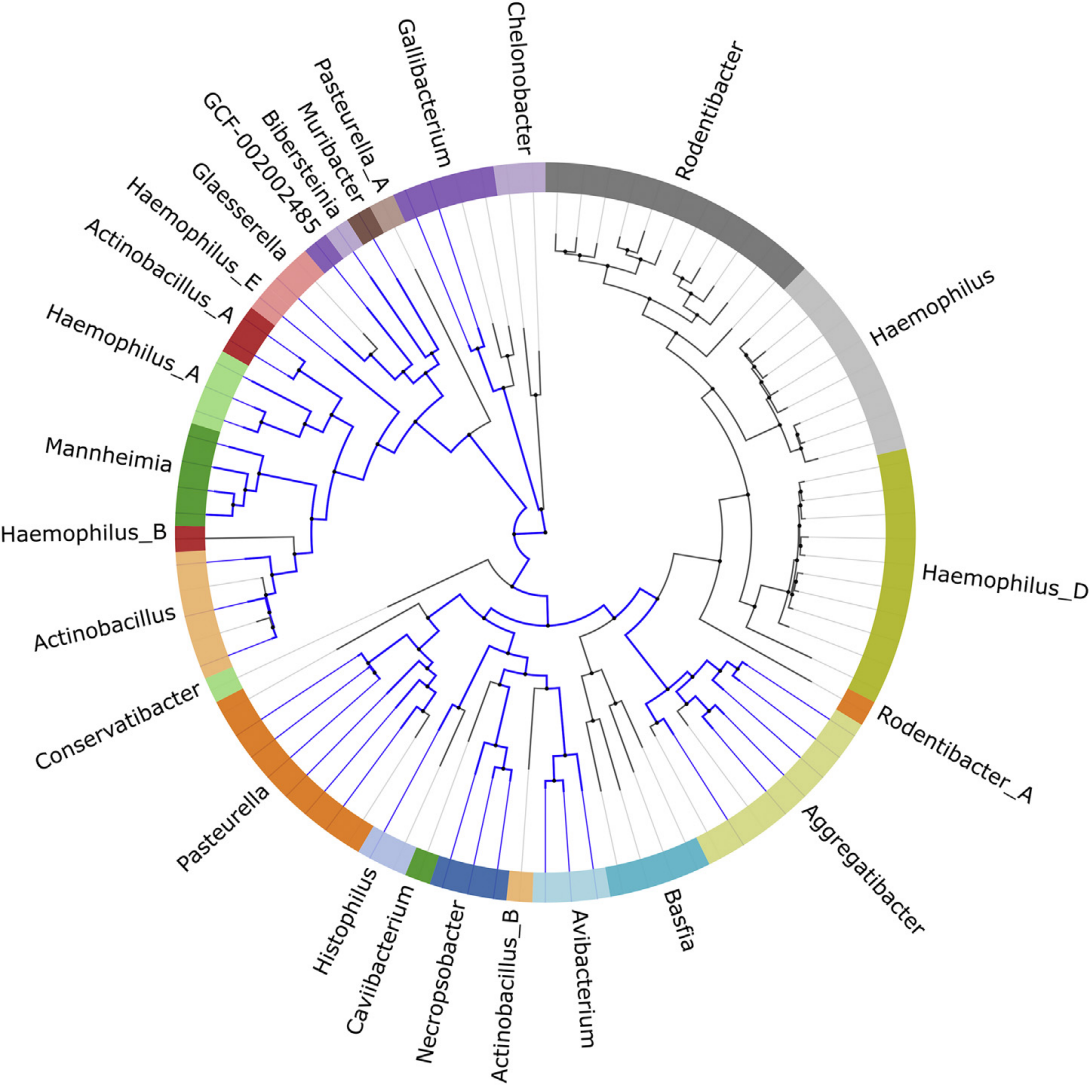
**P5PA prevalence in the Pasteurellaceae.** This tree shows all the species in the family Pasteurellaceae, with the genera labeled and indicated by the colors of the ring. The *blue sections* of the tree indicate those 38 species containing a P5PA protein as identified by our reciprocal BLAST approach. The tree was produced using AnnoTree (version 1.2) (48). P5PA, P5P binding protein A.

From our genomic analysis, we demonstrated that P5PA homologs are exclusively found within the family Pasteurellaceae. The Pasteurellaceae family is predominantly composed of commensal Gram-negative bacteria capable of colonizing the mucosal surfaces of mammals and birds (20, 21). P5PA homologs are exclusively present in the pathogenic strains of the Pasteurellaceae family (Fig. 6). The pathogens containing P5PA are closely associated with respiratory diseases and systemic infections in swine (*Actinobacillus* (22)), rabbits, hares, ruminants (*Actinobacillus* (23), *Mannheimia* (24) and *Pasteurella* (25, 26)), mice (*Muribacter* (27)), and birds (*Pasteurella* (25, 26) and *Gallibacterium* (28)). Moreover, P5PA-encoding bacteria in the genus *Aggregatibacter* are human pathogens and implicated as the dominant cause of infective endocarditis, brain abscesses (*A. aphrophilus*), and periodontal diseases (*Aggregatibacter actinomycetemcomitans*) (29, 30). Taken together, most of the pathogens in the bacterial family Pasteurellaceae possess the P5PA vitamin B_6_ uptake system.

## Discussion

Identified as a homolog of the sugar-phosphate transporter AfuA in *A. pleuropneumoniae*, the functional role of P5PA until now remained unknown. It was hypothesized that P5PA could bind to sugar phosphates just as AfuA because of the significant sequence similarity between the two proteins. Another hypothesis was that P5PA could have been the periplasmic binding protein that binds ferric iron as this has not been identified in *A. pleuropneumoniae* to date and represents an important target as it would traffic the essential nutrient required by bacteria for host colonization. In this study, we structurally characterized P5PA and found it to be a type II periplasmic binding protein capable of binding the active form of vitamin B_6_ (P5P). We confirmed by ITC, MST, and LC–MS/MS that the interaction between P5PA and P5P is highly specific. Moreover, by expressing P5PAB together with the ATPase AfuC from the AfuABC transporter, we were able to rescue the growth of *E. coli* K-12 Δ*pdxB* mutants.

Vitamin B_6_ transport systems in prokaryotes have been poorly characterized. Prior to this study, a very limited number of proteins were disclosed for vitamin B_6_ binding. PdxU1 and PdxU2 were predicted to bind a vitamer of vitamin B_6_. This vitamin is called pyridoxine, and this prediction was not supported by any experimental data (4). YggS was another vitamin B_6_ binding protein structurally identified in *E. coli*, *Saccharomyces cerevisiae*, and *Synechococcus elongatus*. However, the biochemical function of this protein is still undefined (31). In this study, we illustrate that P5PA transports P5P, making it the first reported transporter for the active form of vitamin B_6_ in prokaryotes.

Bacteria have two independent *de novo* pathways to synthesize the active form of vitamin B_6_. Most γ-proteobacteria, including *E. coli*, exploit the deoxyxylulose 5-phosphate (DXP)–dependent pathway where d-erythrose 4-phosphate undergoes a series of biochemical reactions, the product of which then interacts with DXP to form the P5P precursor, pyridoxine-5′-phosphate. This reaction is catalyzed by two key enzymes, PdxA and PdxJ (6, 7). Pyridoxine-5′-phosphate is then converted to pyridoxial-5-phosphate (P5P, active form of vitamin B_6_) *via* a salvage pathway. On the other hand, the DXP-independent pathway is found in the majority of bacteria, and also in archaea, fungi, and plants. In this pathway, vitamin B_6_ is derived from glutamine, ribose 5-phosphate, and glyceraldehyde 3-phosphate with the help of two critical enzymes, PdxS (also referred to as Pdx1) and PdxT (also referred to as Pdx2) (6, 32).

The DXP-independent pathway was recently characterized in *A. pleuropneumoniae* by Xie *et al.* (33). When the authors disrupted the *pdx*S and *pdx*T genes in *A. pleuropneumoniae*, the resulting mutants showed a significant decrease in growth in the absence of P5P. However, supplementation with 10 μM P5P rescued growth (33). These data agree with our observation that there is no significant difference in growth between WT *E. coli* K-12 and the Δ*pdxB* mutant at 5 and 10 μM P5P. This may indicate that, at a high concentration (≥5 μM), exogenous P5P can diffuse into bacterial cells *via* passive transport. In fact, previous studies have illustrated that other dephosphorylated vitamers of vitamin B_6_ enter *E. coli* K-12 and other bacteria by facilitated diffusion (34, 35).

The vitamin B_6_ concentration in swine plasma is maintained at a low level of 36 to 45 nM (36), which suggests that passive movement of vitamin B_6_ across the bacterial membrane is inefficient in the swine host. Hence, possessing a nutrient transporter specific for the active form of vitamin B_6_ increases the chance of successful colonization by *A. pleuropneumoniae* when its vitamin B_6_ biosynthesis pathway is dysfunctional because of deficiencies in starting materials for vitamin B_6_ synthesis. This hypothesis was supported in the present study by the observed effect of expressing the P5PAB together with AfuC in Δ*pdxB* mutants. The relatively high P5P concentrations that were required for *E. coli* expressing *p5pAB + afuC* compared with the concentrations of P5P found in swine blood may reflect P5P exhaustion in the *E. coli* derivative that is completely devoid of vitamin B_6_ production. The P5PA *in vitro* binding affinity for P5P (36 nM) is near the reported physiological levels of P5P in swine blood supporting the beneficial role of this transporter in high-affinity uptake of the active form of vitamin B_6_. Another possible reason for expressing a vitamin B_6_-specific transporter is that nutrient transport is favored over nutrient biosynthesis by microorganisms, since the former consumes less energy (4). In addition to vitamin B_6_, other B-type vitamins are also subject to extracellular uptake by transporters in the ABC transporter family. For example, the important cofactor vitamin B_12_ (cobalamin) can be taken up from the extracellular environment in *E. coli* by the ABC importer BtuCDF and synthesized by the enzymes encoded in the *cod* operon (4, 37). Overcoming nutrient scarcity using these specific transport systems permits bacteria not only to strive in a variety of inhospitable environments but also to outcompete neighboring bacteria for key nutrients including vitamins.

In addition to the critical role in the growth of *A. pleuropneumoniae*, vitamin B_6_ also impacts the morphology of *A. pleuropneumoniae*. The vitamin B_6_ auxotrophs of *A. pleuropneumoniae* were unable to maintain their normal coccobacilli morphology exhibiting holes and craters on the bacterial surfaces while their morphology was not disturbed in the presence of the supplemented vitamin B_6_ (33). Impaired bacterial surfaces may lead to the decreased cell adhesion of *A. pleuropneumoniae* to host cells, which is the first step of host colonization. Furthermore, the reduced colonization load and attenuated virulence were observed in mice inoculated with the vitamin B_6_ auxotrophs of *A. pleuropneumoniae* (33) indicating vitamin B_6_ plays an important role in the virulence of *A. pleuropneumoniae*. As a transporter of vitamin B_6_, P5PA may also have an influence on the virulence of *A. pleuropneumoniae* making it a potential novel drug target for *A. pleuropneumoniae* infection treatment. Moreover, as P5PA vitamin B_6_ uptake systems are widely conserved in pathogens within the Pasteurellaceae family, it is possible that P5PA may contribute by providing a fitness advantage to allow this family to persist in natural flora. However, further experiments on ΔP5PA mutants of *A. pleuropneumoniae* are needed to delineate the roles of this gene in virulence.

## Experimental procedures

### Bacterial strains and growth conditions

All bacterial strains used in this study are listed in Table S1. All *E. coli* strains were grown in LB broth or M9 minimal media (Bioshop) or on LB agar plates at 37 °C. When antibiotics were needed for plasmid selection, they were added at the following concentrations: 50 μg/ml kanamycin and 50 μg/ml ampicillin.

### Cloning and expression of P5PA, P5PB (APJL_RS02705), and AfuC

Plasmids used in this study are listed in Table S1. To construct pET26b-HisP5PA, the coding sequence of P5PA was amplified from the genomic DNA of *A. pleuropneumoniae* strain H49 and integrated into a pET26b expression vector by restriction-free cloning (38). To construct the plasmids pSC101-P5PAB and pSC101-P5PAB + AfuC, the operon containing *p5pAB* was cloned into the low-copy customized vectors pSC101 and pSC101-AfuABC by exponential megaprimer cloning (39). pSC101-AfuABC was obtained from the previous study performed by Sit *et al.* (15). Site mutagenesis was utilized to introduce desired mutations of P5PA to the parental plasmids pET26-HisP5PA and pSC101-P5PAB + AfuC (15). All constructed plasmids were verified by TCAG DNA sequencing (The Centre for Applied Genomics).

### P5PA protein purification

The plasmid pET26b-HisP5PA was transformed into *E. coli* BL21 competent cells, which were then grown overnight in LB broth supplemented with 50 μg/ml kanamycin at 37 °C with shaking. The overnight culture was then used to inoculate 2YT media (1:100) for large-scale protein expression. IPTG was added to induce protein overexpression with a concentration of 0.5 mM when an absorbance reached 0.6 at 600 nm. After 4 h of incubation at 20 °C, cells were harvested by centrifugation at 7500 rpm at 4 °C and resuspended in lysis buffer (10 mM imidazole, 50 mM Tris, pH 8, and 300 mM NaCl) containing 1 mM PMSF, 1 mM benzamidine, 1 mg/ml lysozyme, and 0.05 mg/ml DNases. The cell suspension was sonicated for 15 min with 15 s ON/30 s OFF intervals to lyze the cells. This was followed by centrifugation at 17,000 rpm for 30 min to pellet cell debris (40). The supernatant was passed through a 0.45 μm syringe filter, combined with 1 ml of Ni–NTA resin, and incubated at 4 °C for at least 1 h. The solution was then loaded into a gravity column, washed with lysis buffer, and eluted with elution buffer (400 mM imidazole, 50 mM Tris, pH 8, and 300 mM NaCl). Subsequently, the eluted protein was dialyzed with thrombin in the lysis buffer to cleave off 6×His tag from the N terminus of P5PA. After overnight dialysis at 4 °C, Ni–NTA resin and benzamidine resin were used to remove uncleaved His-P5PA, thrombin, and the free 6×His tag. The protein solution was then centrifuged at 13,000 rpm for 10 min and concentrated in a centrifugal concentrator with a 10 kDa cutoff (Millipore). Finally, the protein was loaded onto a Superdex 75 10/300 gel filtration column (GE Healthcare), which had been equilibrated in buffer containing 20 mM Tris, pH 8.0 and 100 mM NaCl, for further purification (15). The purity and yield of protein was estimated by SDS-PAGE, and 280-nm readings were made on a Nanodrop, respectively. The N-terminal 6×His tag was only cleaved for P5PA used in X-ray crystallography trials.

### Crystallization, data collection, and structure determination

P5PA (20 mM Tris, pH 8, and 100 mM NaCl) was concentrated to 12 mg/ml and used in screen-based sitting-drop crystallization trials. The Gryphon robot (Art Robbins Instrument) was used to set up the MSCG 1 to 4, JCSG1, and index commercial screens with drop sizes of 0.4 μl. P5PA was initially crystallized in two hit conditions: 0.1 M potassium thiocyanate, 30% PEG MME 2000; 0.1 M Ches: NaOH, pH 9.5, and 30% PEG 3000. The crystal hits were manually optimized in a 1:1 volume ratio of protein:precipitant by varying the pH and the concentrations of PEG and glycerol. Candidate crystals were looped from the condition (0.05 M potassium thiocyanate and 27.5% PEG MME 2000) into a cryoprotectant solution consisting of the mother liquor with 20% glycerol and flash frozen in liquid nitrogen. The crystals were sent to the Advanced Photon Source Synchrotron Facility (Argonne) for data collection. MR was performed by running Phaser in PHENIX (41) using AfuA (Protein Data Bank [PDB] ID: 4R73) as the template (54% sequence identity shared with P5PA). MR and automated refinement were performed using PHENIX. Manual refinement was performed using Coot (42). Molecular models (Fig. 1) were generated using PyMol (The PyMOL Molecular Graphics System, version 2.0; Schrödinger, LLC).

### ITC

The purification method described in the P5PA protein purification section was modified as follows in order to purify apo-P5PA for ITC. Before elution, P5PAwas denatured in the gravity column. To denature P5PA, 6 M guanidine hydrochloride (GdHCl) was applied to the column, after which a refolding step was performed by stepwise addition of 4.5 M GdHCl, 3 M GdHCl, and finally 1.5 M GdHCl. The protein was then washed with lysis buffer and eluted with elution buffer (15). The subsequent purification steps were the same as previously described.

ITC was performed using an auto-iTC_200_ (Microcal) at the Structure and Biophysical Core Facility within the Peter Gilgan Centre for Research & Learning (Hospital for Sick Children, Toronto, Canada). Runs consisted of titrating ligand loaded into the injection syringe against purified protein, which was loaded into the sample cell. About 45 μM of purified P5PA was stored in ITC buffer (20 mM Tris, pH 8, and 100 mM NaCl) at 4 °C. The same buffer was used to make solutions of 300 μM G6P, 300 μM P5P, and 45 μM bovine serum albumin. P5PA, bovine serum albumin, and ITC buffer alone were titrated with P5P or G6P. The parameter setting was 2 μl/injection for a total of 20 injections. Data applied to calculate binding constants were referenced against runs performed with ligand alone to control for the heat of ligand solvation.

### P5PA fluorescent labeling and MST

P5PA was fluorescently labeled using RED-NHS Second Generation Protein Labeling Kit (NanoTemper). Briefly, 90 μl of purified apo-P5PA at 10 μM was mixed with 10 μl of the provided labeling dye at 300 μM. The mixture was incubated in dark for 30 min and loaded to a special gravity column that is pre-equilibrated in PBS with 0.05% Tween-20 (PBST). After washing the column with 550 μl of PBST, another 450 μl of PBST was added to elute the fluorescently labeled apo-P5PA. The yield of the labeled P5PA was determined by 280-nm readings made on a Nanodrop.

A target compound was successively diluted in PBST by a dilution factor of two to make a total of 16 different concentrations. Then, 10 μl of the labeled P5PA was added to an equal volume of the diluted compounds and loaded to standard capillaries (NanoTemper). The Binding Affinity Mode of Monolith NT.115 (NanoTemper) was used to detect fluorescence changes and deduct *K*_*d*_. The corresponding final concentrations of the labeled P5PA and the tested compounds were listed in Table S2.

### Identification of the P5PA ligand by LC–MS/MS

Endogenous ligands of P5PA were sought by liquid–liquid extraction. About 1 ml of 80% cold acetone (−20 °C) and 20% cold 2 M HCl was added to P5PA in ITC buffer. The mixture was then vortexed for 5 min, incubated at 4 °C overnight, and centrifuged at 15,000 rpm for 10 min. The supernatant was harvested and dried by SpeedVac SC100 (Savant). The dried pellet was dissolved in milliQ water, and the solution was then used for liquid–liquid extraction with acetonitrile and saturated NH_4_Cl to remove excess NaCl and Tris from the solution (43, 44). The organic layer was transferred to a new tube and dried. The dried precipitates were dissolved in milliQ water and sent to the Analytical Facility for Bioactive Molecules (Hospital for Sick Children) for LC–MS/MS.

### *E. coli* growth assays

The Δ*pdxB* mutant of *E. coli* K-12 strain was obtained from the Keio collection (45). The Δ*pdxB* mutant was transformed with either pSC101, pSC101-P5PAB, or pSC101-P5PAB + AfuC and grown in LB broth overnight at 37 °C with shaking. The starters were then washed twice with M9 medium and inoculated at an absorbance of 0.05 at 600 nm into M9 medium supplemented with P5P. The P5P concentrations used were 0, 1, 2.5, and 5 μM. Growth assays were performed in both culture tubes and 100-well microplates (15, 17). For cell growth in culture tubes, 5 ml of P5P-supplemented M9 was used, and readings of absorbance at 600 nm were measured after a 23-h incubation at 37 °C with shaking. For cell growth in 100-well microplates, 200 μl of P5P-supplemented M9 was applied to each well, and microplates were incubated in a Bioscreen C microplate reader (Growth Curves USA) at 37 °C with shaking. Absorbance values at 600 nm were measured every 15 min for a time course of 40 h.

Δ*pdxB* mutant complemented with pSC101-P5PA_(Y103A)_B + AfuC, pSC101-P5PA_(T149A)_B + AfuC, and pSC101-P5PA_(H203A)_B + AfuC were grown using the same conditions with minor modifications. A 96-well microplate was used and incubated in a Cytation five multimode reader (Biotek USA) with shaking. Values of absorbance at 600 nm were measured every 15 min for a time course of 20 h.

### *In silico* detection of P5PA

As P5PA and AfuA share significant sequence similarity (54% amino acid identity), a reciprocal BLAST approach was used specifically to identify P5PA homologs and discriminate from AfuA. We first queried a P5PA protein sequence (accession: WP_012262855) against the nr database using BLASTp (v2.10.0+) (46). The top 100 hits shared >66% identity, so an additional seven sequences were chosen as a set of “seed sequences” for which we had high confidence in their function. These eight seed sequences were then queried against the refseq protein database using BLASTp, excluding hits from all eight seed species. This returned 64 hits that shared >65% identity over >80% of the sequence with at least one seed sequence. Nine hits showed similarity to only one or two seed sequences and so were added to the set of seed sequences to improve our detection ability. These 17 seed sequences were then queried against the refseq_protein database using BLASTp, again excluding all hits from the seed species. This returned 88 hits sharing >65% identity over >80% of the sequence.

A similar process was used to identify eight AfuA seed sequences, which were queried against the refseq protein database using BLASTp, and returned 234 hits sharing >65% identity over >80% of the sequence. The 88 putative P5PA hits were then queried against the set consisting of 17 P5PA seed sequences, eight AfuA seed sequences, and 234 AfuA sequences using BLASTp. P5PA hits were only considered valid if all three of the most similar sequences were from the P5PA seed sequences and not from the set of AfuA sequences. All 88 hits passed this threshold, and so when combined with the seed sequences, we were able to identify a total of 105 P5PA sequences. In order to further differentiate the sequences with higher confidence, we used the “differential enrichment” mode of MEME (47) to identify sequence motifs present in P5PA that are as different as possible from AfuA. We found a set of ten motifs demonstrating a consistent pattern in all P5PA sequences.

## Data availability

The PDB coordinates for the P5PA structure have been deposited with PDB accession code: 6WCE. All other data are presented within the article or supporting information.

## Supporting information

This article contains supporting information (49, 50).

## Conflict of interest

The authors declare that they have no conflicts of interest with the contents of this article.
