# Supplementary material for: Actinobacillus utilizes a binding protein–dependent ABC transporter to acquire the active form of vitamin B6
Source: J Biol Chem. 2021 Aug 4;297(3):101046. doi: 10.1016/j.jbc.2021.101046 (PMC8427247; doi:10.1016/j.jbc.2021.101046)
Supplement: Supplemental Figures S1–S7 and Tables S1–S2 [file mmc1.pdf]

Supplementary Information

Actinobacillus utilizes a binding-protein dependent ABC transporter to acquire the active form of vitamin B_6

Chuxi Pan[a], Alexandra Zimmer[a], Megha Shah[a], Minh Sang Huynh, Christine Chieh-Lin Lai[a], Brandon Sit[a], Yogesh Hooda[a], David M. Curran[a] and Trevor F. Moraes[a,*]

There are 7 supplementary figures and 2 supplementary table associated with this manuscript.

Supplementary Table1. Strains and plasmids used in this study

Strain or plasmid	Description
**Strains**	
Escherichia coli BL21	F⁻ *ompT gal dcm lon* $hsdS_B(r_B^- m_B^-)$ λ(DE3 [*lacI lacUV5-T7p07 ind1 sam7 nin5*]) [$malB^+$]$_{K\text{-}12}$(λ^S)
Escherichia coli K-12 BW25113	$lacI^+ rrnB_{T14}$ Δ$lacZ_{WJ16}$ *hsdR*514 Δ$araBAD_{AH33}$ Δ$rhaBAD_{LD78}$ *rph-1* Δ*(araB–D)567* Δ*(rhaD–B)568* Δ*lacZ4787*(::*rrnB-3*) *hsdR514 rph-1*
Escherichia coli K-12 Δ*pdxB*	$lacI^+ rrnB_{T14}$ Δ$lacZ_{WJ16}$ *hsdR*514 Δ$araBAD_{AH33}$ Δ$rhaBAD_{LD78}$ *rph-1* Δ*(araB–D)567* Δ*(rhaD–B)568* Δ*lacZ4787*(::*rrnB-3*) *hsdR514 rph-1* Δ*pdxB729*::*kan*
**Plasmids**	
pET26-HisP5PA	Expression vector for *A. pleuropneumoniae* wild type N-terminal histidine-tagged P5PA; Kan^R
pET26-HisP5PA-Y103A	Expression vector for *A. pleuropneumoniae* mutated N-terminal histidine-tagged P5PA-Y103A; Kan^R
pET26-HisP5PA-T149A	Expression vector for *A. pleuropneumoniae* mutated N-terminal histidine-tagged P5PA-T149A; Kan^R
pET26-HisP5PA-H203A	Expression vector for *A. pleuropneumoniae* mutated N-terminal histidine-tagged P5PA-H203A; Kan^R
pSC101	Custom vector with pSC101 ori; Amp^R
pSC101-P5PAB	pSC101 vector + *A. pleuropneumoniae* P5PAB with -226bp upstream; Amp^R
pSC101-P5PAB+AfuC	pSC101 vector + *A. pleuropneumoniae* P5PAB+AfuC with -226bp upstream; Amp^R
pSC101-P5PA$_{(Y103A)}$B+AfuC	pSC101 vector + *A. pleuropneumoniae* P5PAB+AfuC with -226bp upstream; Amp^R
pSC101-P5PA$_{(T149A)}$B+AfuC	pSC101 vector + *A. pleuropneumoniae* P5PAB+AfuC with -226bp upstream; Amp^R
pSC101-P5PA$_{(H203A)}$B+AfuC	pSC101 vector + *A. pleuropneumoniae* P5PAB+AfuC with -226bp upstream; Amp^R
pSC101-APAfuABC	pSC101 vector + *A. pleuropneumoniae* AfuABC with -262bp upstream; Amp^R

Supplementary Table2. The concentrations of protein and compounds used in MST

Labeled Protein	Concentration (nM)	Compound	Highest Working Concentration
**P5PA**	25	**Pyridoxal-5'-phosphate (P5P)**	1.40 μM
**P5PA-Y103A**	50	**Pyridoxal-5'-phosphate (P5P)**	1.75 mM
**P5PA-T149A**	25	**Pyridoxal-5'-phosphate (P5P)**	0.35 mM
**P5PA-H203A**	100	**Pyridoxal-5'-phosphate (P5P)**	3.50 mM
**P5PA**	25	**Pyridoxal (PL)**	448 mM
**P5PA**	25	**Pyridoxamine (PM)**	448 mM
**P5PA**	25	**Glucose-6-phosphate (G6P)**	448 mM

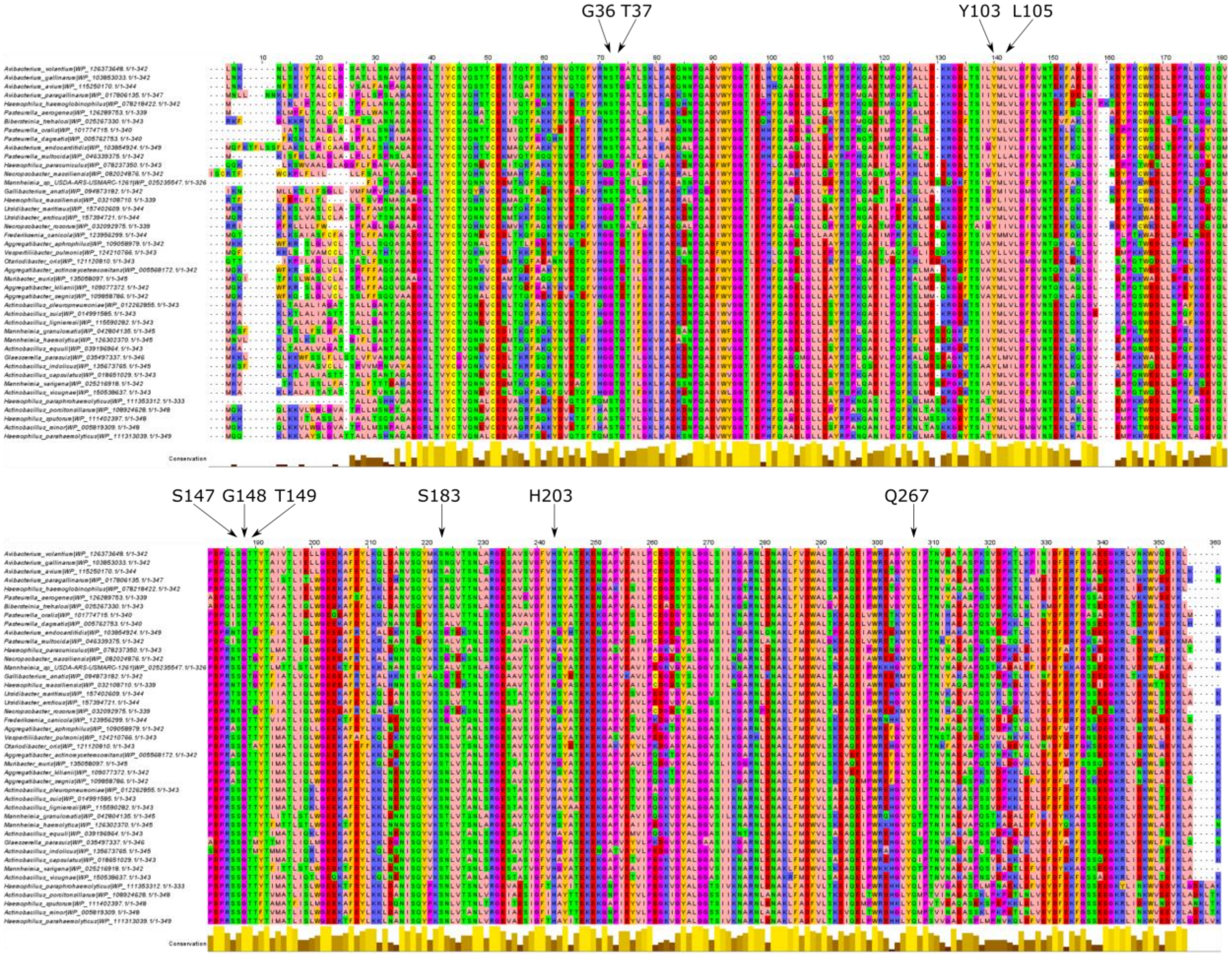

Supplementary Figure 1. Multiple sequence alignment of putative P5PA sequences. Residues are colored according to their chemical properties, and the black arrows indicate regions from the A. pleuropneumoniae protein P5PA sequence discussed in the text. The alignment was generated using the G-INS-i algorithm from MAFFT (v7.450)(49), and has been visualized using JalView (v2.11.0)(50).

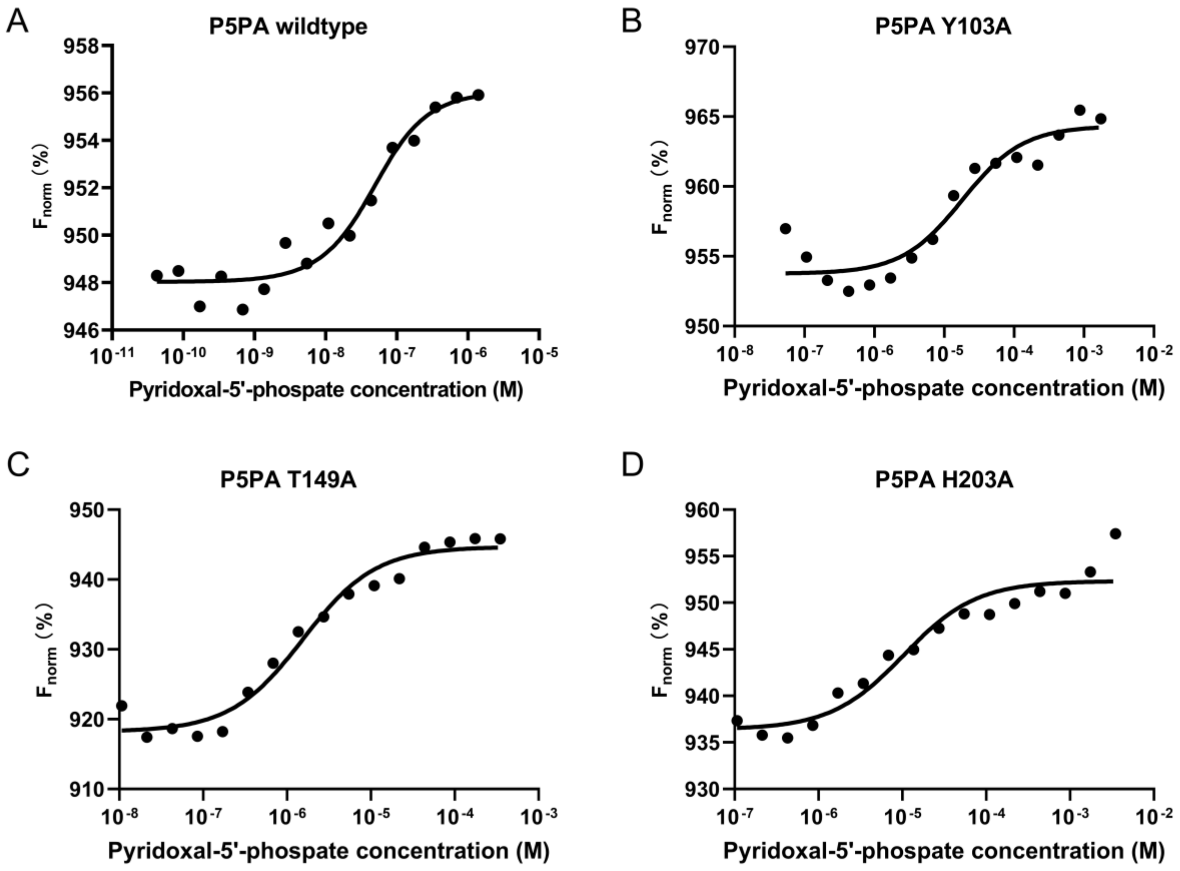

Supplementary Figure 2. P5PA mutants did not lose their P5P-binding capacities. **(A)** Representative MST data showing the binding curve between wild-type P5PA and P5P. 25 nM fluorescence-labelled wild-type P5PA was mixed with a serial dilution of P5P starting at 1.4 μM. The reaction mixtures were heated and recorded by MST. **(B)** Representative MST data showing the binding curve between P5PA Y103A mutant and P5P. 50 nM fluorescence labelled P5PA Y103A mutant was mixed with a serial dilution of P5P starting at 1.75 mM. The rest parameters were the same as (A). **(C)** Representative MST data showing the binding curve between P5PA T149A mutant and P5P. 25 nM fluorescence labelled P5PA T149A mutant was mixed with a serial dilution of P5P starting at 350 μM. The rest parameters were the same as (A). **(D)** Representative MST data showing the binding curve between P5PA H203A mutant and P5P. 100 nM fluorescence labelled P5PA T149A mutant was mixed with a serial dilution of P5P starting at 3.5 mM. The rest parameters were the same as (A).

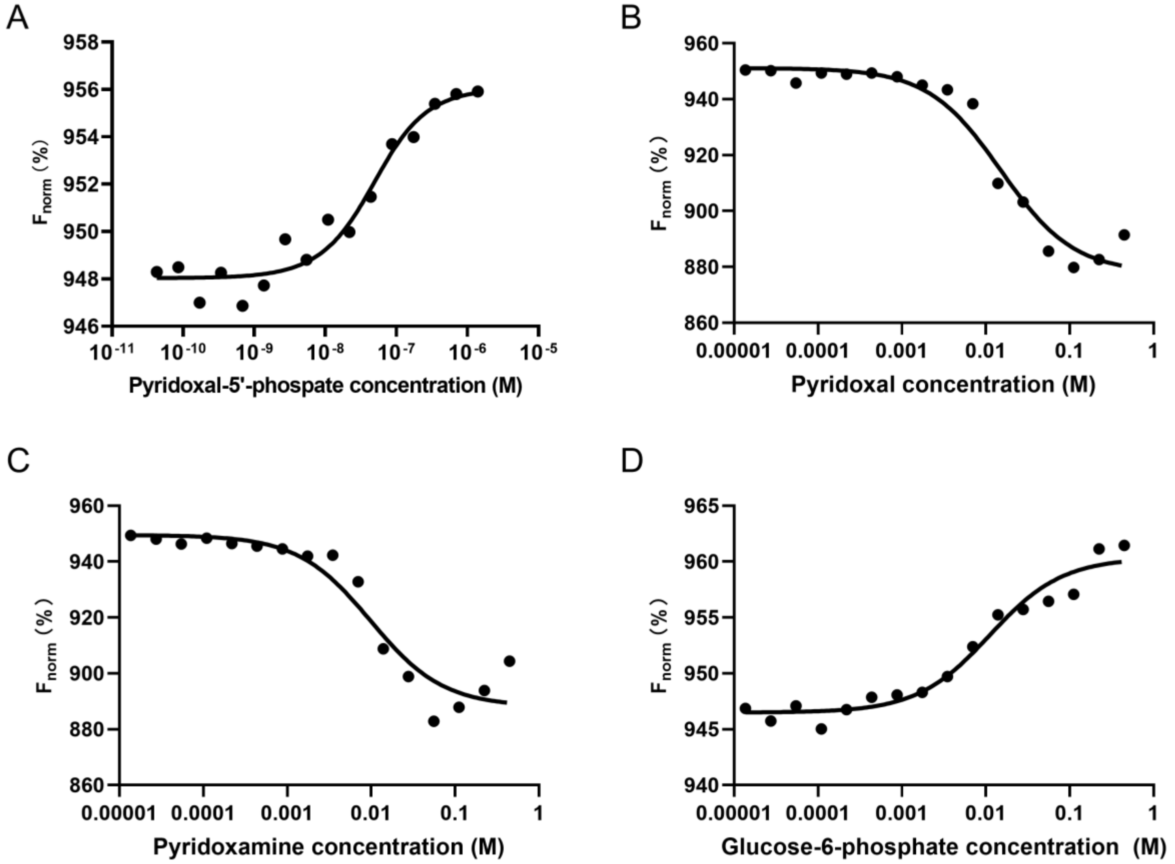

Supplementary Figure 3. P5PA non-specifically interacted with two vitamin B_6 vitamers PM and PL at 448 mM. **(A)** Representative MST data showing the binding curve between P5PA and P5P as shown in Supplementary Figure 2A above. 25 nM fluorescence-labelled wild-type P5PA was mixed with a serial dilution of P5P starting at 1.4 μM. The reaction mixtures were heated and recorded by MST. **(B)** Representative MST data showing the binding curve between P5PA and pyridoxal. 25 nM fluorescence labelled P5PA was mixed with a serial dilution of pyridoxal starting at 448 mM. The rest parameters were the same as (A). **(C)** Representative MST data showing the binding curve between P5PA and pyridoxamine. All parameters were the same as (B). **(D)** Representative MST data showing the binding curve between P5PA and G6P. All parameters were the same as (B).

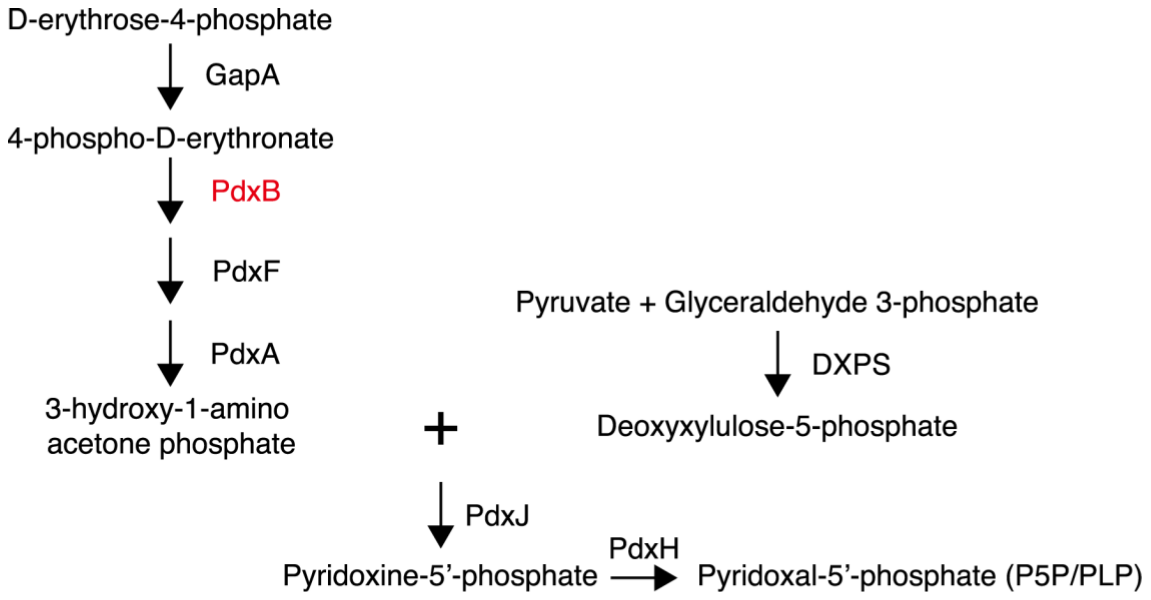

Supplementary Figure 4. Vitamin B_6 de novo biosynthesis pathway in *E. coli* K-12. In *E. coli* K-12, D-erythrose-4-phosphate, pyruvate and glyceraldehyde-3-phosphate can be used to synthesize P5P. The synthesis is catalyzed by GapA, DXPS and enzymes encoded by the pdx genes. Each arrow represents an enzymatic reaction between a pathway intermediate and the enzymes alongside the arrows.

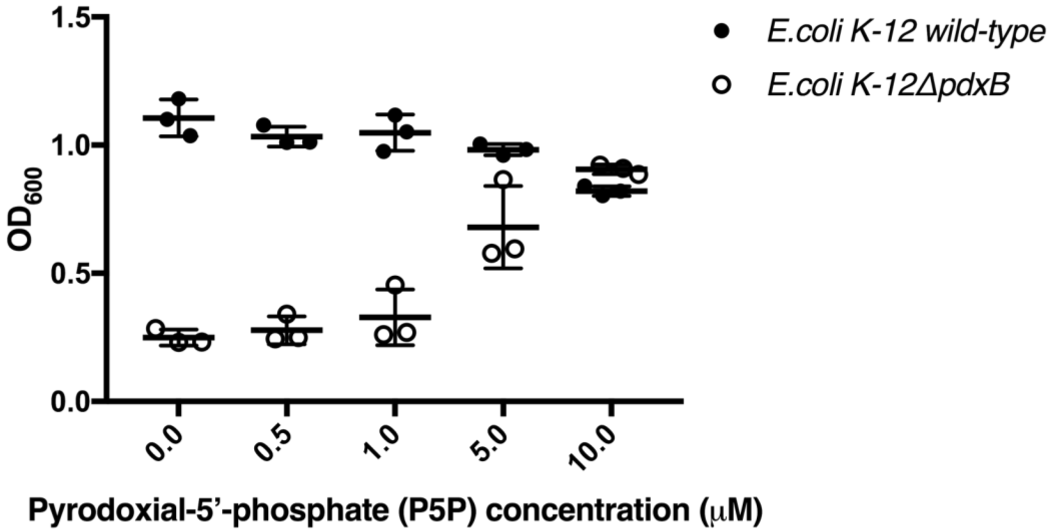

Supplementary Figure 5. *E. coli* K-12 Δ*pdxB* mutant has a growth deficiency in M9 supplemented with vitamin B_6 (Pyrodoxial-5'-phosphate -P5P) at 0.5 µM and 1 µM. With starting OD_{600}=0.05, *E. coli* K-12 wild-type and Δ*pdxB* mutant were incubated in 5 mL of M9 supplemented with vitamin B_6 at a series concentration of 0.5 µM, 1 µM, 5 µM and 10 µM. OD_{600} was measured after a 23 hour incubation at 37°C.

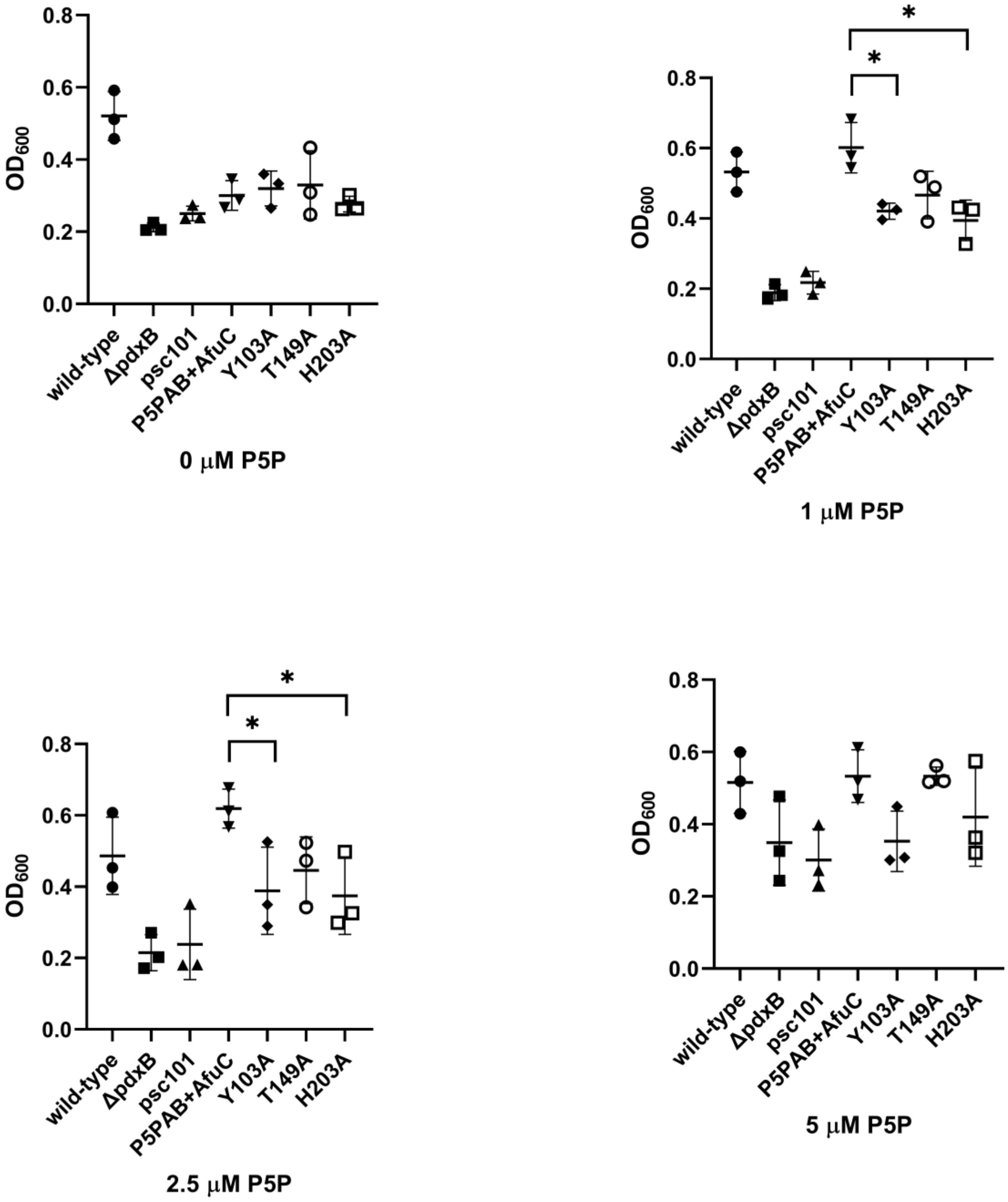

Supplementary Figure 6. *p5pAB+afuC* mutants restored the growth rate of *E. coli* K-12 Δ*pdxB* mutant. *E. coli* K-12 Δ*pdxB* mutants were transformed with four constructs: pSC101, pSC101-P5PAB+AfuC, pSC101- P5PAB+AfuC-Y103A, pSC101- P5PAB+AfuC-T149A and pSC101- P5PAB+AfuC-H203A. They were then incubated in 200 µL of M9 supplemented with vitamin B_6 at 0 µM**,** 1 µM, 2.5 µM and 5 µM. The starting OD_{600} was 0.03 and OD_{600} reading was measured after 20 hours. The error bars represent standard deviations from n=3 transformants. (*P<0.05)

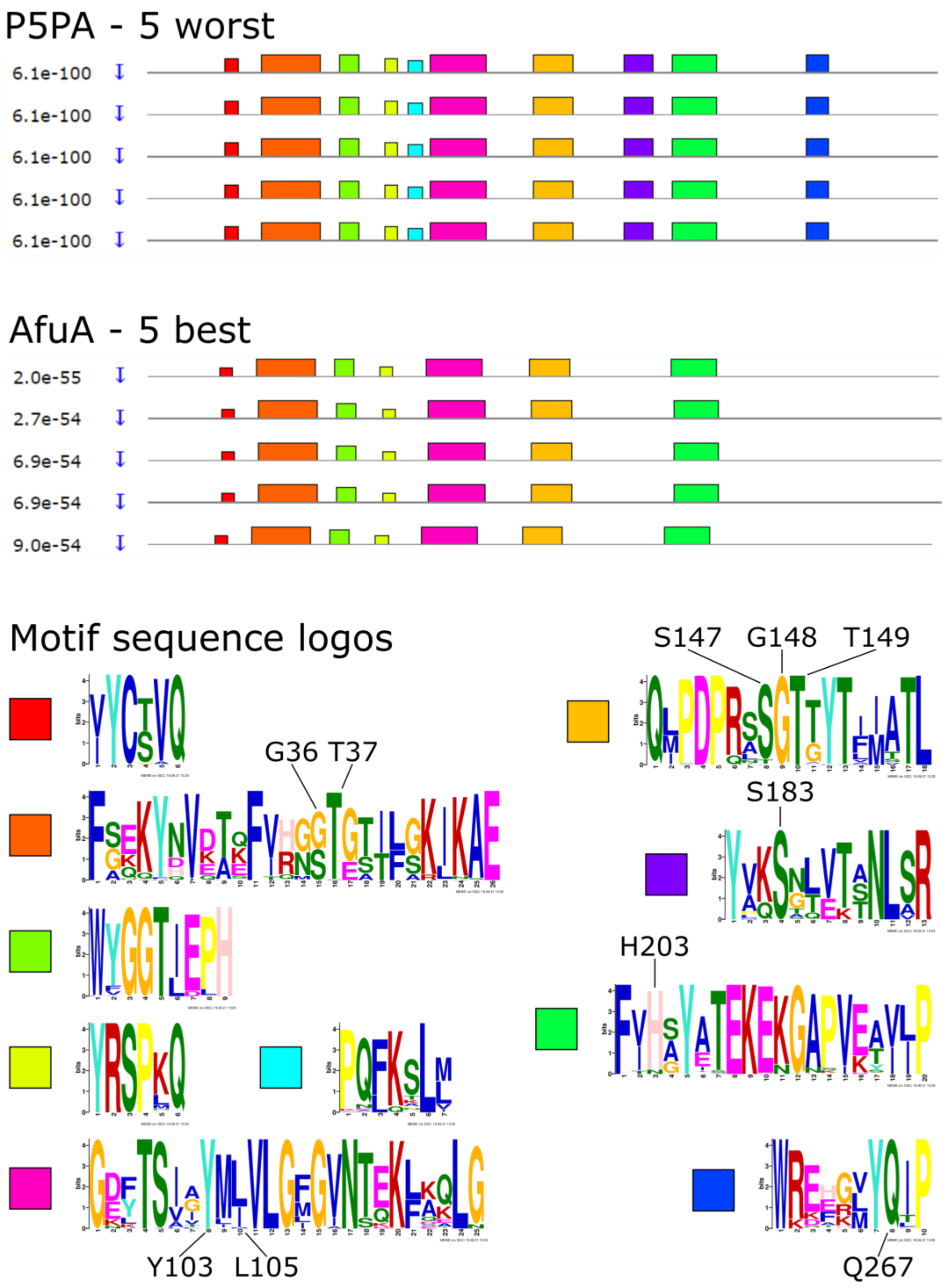

Supplementary Figure 7. The P5PA sequence motifs identified using MEME. Each horizontal line indicates one sequence, where the colored boxes indicate the location that a particular motif matches, and the height of the box indicates how well that motif matches to the given sequence. The E-value is a product of the strength of the individual motif matches. All P5PA sequences had E-values <6.1E-100 and the full complement of 10 motifs. All AfuA sequences had substantially worse E-values (none were <2E-55), only approximately 1/3 of the sequences had a weak match to the 4th (yellow) or the 10th (blue) motif, and none matched the 8th (purple) motif. This shows that these 10 motifs provide a clear way to differentiate P5PA and AfuA, both by E-value and by motif complement.
